# Supplementary material for: Construction of Hollow Co3O4@ZnIn2S4 p-n Heterojunctions for Highly Efficient Photocatalytic Hydrogen Production
Source: Nanomaterials (Basel). 2023 Feb 17;13(4):758. doi: 10.3390/nano13040758 (PMC9960535; doi:10.3390/nano13040758)

Table S1. Comparison with other catalysts

Semiconductor	Solvothermal method	Morphology	Sacrificial reagent	Light source (nm)	Optimal hydrogen evolution (μmol·g−1·h−1)	Refs.
$ZnIn_2S_4$@ NH_2-MIL-125(Ti)	Solvothermal method	Micropores and mesopores	Na_2S/Na_2SO_3	300 W Xe lamp λ > 420 nm	2204.2 40 wt% NH_2-MIL-125(Ti)	1
g-C_3N_4@ $ZnIn_2S_4$	Solvothermal method	2D/2D g-C3N4 nanosheet@ ZnIn2S4 nanoleaf	Triethanolamine	300 W Xe lamp λ > 420 nm	2780	2
Co(dmgH)$_2$pyC /$ZnIn_2S_4$	Impregnating method	Microspheres	Triethanolamine	300 W Xe lamp λ > 420 nm	3840 3.0 wt% Co(dmgH)$_2$pyCl/ ZnIn2S4	3
CQDs/$ZnIn_2S_4$	Microwave hydrothermal method	Microspheres	N/A	350 W Xe lamp λ > 400 nm	1032.2	4
$MoS_2/ZnIn_2S_4$	Hydrothermal method	Nanoparticles	Na_2S/Na_2SO_3	300 W Xe lamp λ > 420 nm	2080 0.5 wt% $MoS_2/ZnIn_2S_4$	5
$In_2S_3/ZnIn_2S_4$	Ion-exchange method	Microflowers	Na_2S/K_2SO_3	300 W Xe lamp λ ≥ 420 nm	678	6
CdS/$ZnFe_2O_4$/ $ZnIn_2S_4$	Solvothermal processes & Ionic layer adsorption-reaction method	Nanosheet stereoscopic films	Na_2S/Na_2SO_3	300 W Xe lamp λ > 420 nm	79.0 μmol h−1 1-CdS/$ZnFe_2O_4$/ $ZnIn_2S_4$	7

[1] H. Liu, J. Zhang, D. Ao, Construction of heterostructured ZnIn2S4@NH2-MIL-125(Ti) nanocomposites for visible-light-driven H2 production, Appl. Catal. B Environ. 221 (2018) 433–442.

[2] B. Lin, H. Li, H. An, W. Hao, J. Wei, Y. Dai, C. Ma, G. Yang, Preparation of 2D/2D g-C3N4 nanosheet@ZnIn2S4 nanoleaf heterojunctions with well-designed high-speed charge transfer nanochannels towards high-efficiency photocatalytic hydrogen evolution, Appl. Catal. B Environ. 220 (2018) 542–552.

[3] Y. Gao, H. Lin, S. Zhang, Z. Li, Co(dmgH)2pyCl as a noble-metal-free co-catalyst for highly efficient photocatalytic hydrogen evolution over hexagonal ZnIn2S4, RSC

Adv. 6 (2016) 6072–6076.
[4] Q. Li, C. Cui, H. Meng, J. Yu, Visible-light photocatalytic hydrogen production activity of ZnIn2S4 microspheres using carbon quantum dots and platinum as dual co-catalysts, Chem. Asian. J. 9 (2014) 1766–1770.
[5] T. Huang, W. Chen, T. Liu, Q. Hao, X. Liu, Znln2S4 hybrid with MoS2: a non-noble metal photocatalyst with efficient photocatalytic activity for hydrogen evolution, Powder. Technol. 315 (2017) 157–162.
[6] Z. Mei, S. Ouyang, D. Tang, T. Kako, D. Golberg, J. Ye, An ion-exchange route for the synthesis of hierarchical In2S3/ZnIn2S4 bulk composite and its photocatalytic activity under visible-light irradiation, Dalton Trans. 42 (2013) 2687–2690.
[7] Y. Chen, G. Tian, W. Zhou, Y. Xiao, J. Wang, X. Zhang, H. Fu, Enhanced photo-generated carrier separation in CdS quantum dot sensitized ZnFe2O4/ZnIn2S4 nanosheet stereoscopic films for exceptional visible light photocatalytic H2 evolution performance, Nanoscale 9 (2017) 5912–5921.

Figure S1. TG results of the Co_3O_4(20)@ZIS

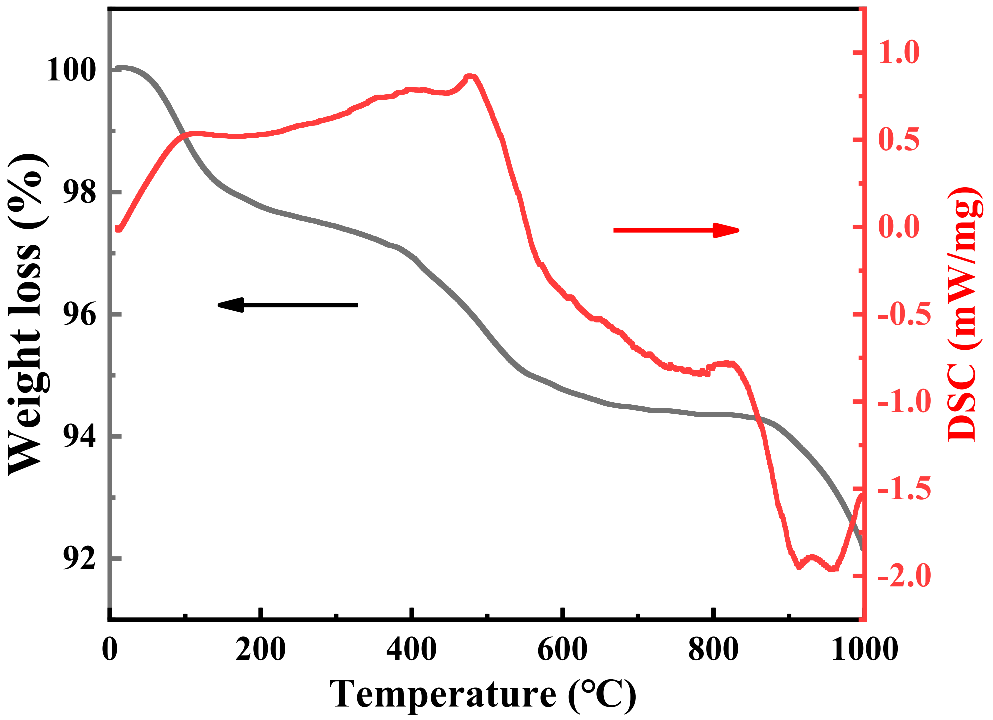

Figure S2. Three cycles of experiment of the Co_3O_4(20)@ZIS

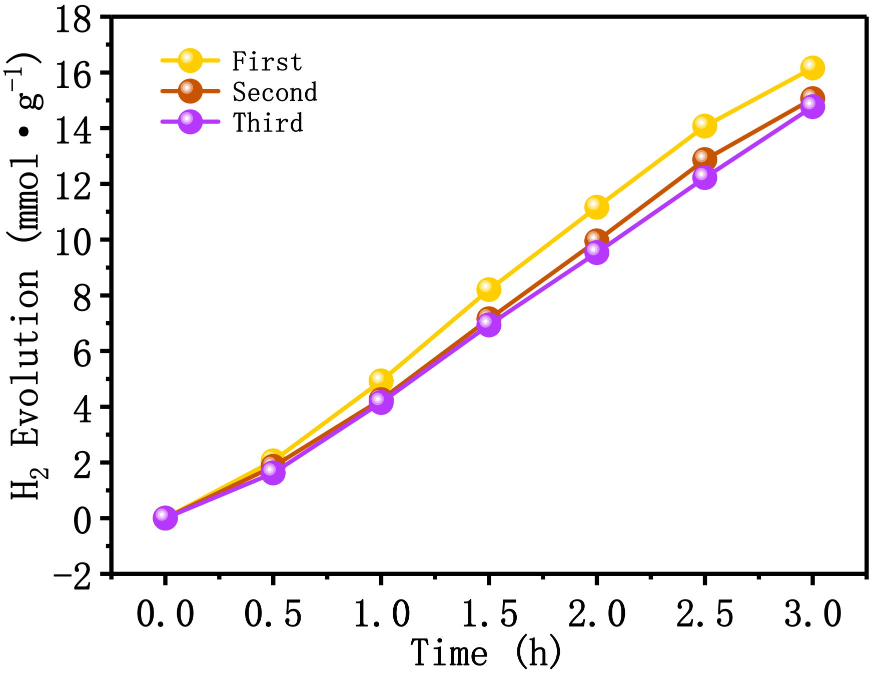

Supplement: Supplementary file 1 [file nanomaterials-13-00758-s001.zip › nanomaterials-2179387-supplementary.pdf]
